# The small molecule tyrosine kinase inhibitor NVP-BHG712 antagonizes ABCC10-mediated paclitaxel resistance: a preclinical and pharmacokinetic study

**DOI:** 10.18632/oncotarget.2638

**Published:** 2014-10-28

**Authors:** Rishil J. Kathawala, Liuya Wei, Nagaraju Anreddy, Kang Chen, Atish Patel, Saeed Alqahtani, Yun-Kai Zhang, Yi-Jun Wang, Kamlesh Sodani, Amal Kaddoumi, Charles R. Ashby, Zhe-Sheng Chen

**Affiliations:** ^1^ Department of Pharmaceutical Sciences, College of Pharmacy and Health Sciences, St. John's University, Queens, NY, USA; ^2^ School of Pharmacy and Biological Sciences, Weifang Medical University, Weifang, People's Republic of China; ^3^ Department of Obstetrics and Gynecology, Wayne State University School of Medicine, Detroit, Michigan, USA; ^4^ Perinatology Research Branch, Eunice Kennedy Shriver National Institute of Child Health and Human Development, National Institutes of Health, Bethesda, Maryland, USA; ^5^ Tumor Biology and Microenvironment Program, Barbara Ann Karmanos Cancer Institute, Detroit, Michigan, USA; ^6^ Mucosal Immunology Studies Team, National Institute of Allergy and Infectious Diseases, National Institutes of Health, Bethesda, Maryland, USA; ^7^ Department of Basic Pharmaceutical Sciences, College of Pharmacy, The University of Louisiana, Monroe, LA, USA; ^8^ Current address: Division of Oncology, Stanford University, Stanford, CA, USA

**Keywords:** NVP-BHG712, ABCC10, ABC transporters, Paclitaxel, Tyrosine kinase inhibitors

## Abstract

Paclitaxel exhibits clinical activity against a wide variety of solid tumors. However, resistance to paclitaxel significantly attenuates the response to chemotherapy. The ABC transporter subfamily C member 10 (ABCC10), also known as multi-drug resistance protein 7 (MRP7) efflux transporter, is a major mediator of paclitaxel resistance. Here, we determine the effect of NVP-BHG712, a specific EphB4 receptor inhibitor, on 1) paclitaxel resistance in HEK293 cells transfected with *ABCC10,* 2) the growth of tumors in athymic nude mice that received NVP-BHG712 and paclitaxel systemically and 3) the pharmacokinetics of paclitaxel in presence or absence of NVP-BHG712. NVP-BHG712 (0.5 μM), in HEK293/ABCC10 cells, significantly enhanced the intracellular accumulation of paclitaxel by inhibiting the efflux activity of ABCC10 without altering the expression level of the ABCC10 protein. Furthermore, NVP-BHG712 (25 mg/kg, p.o., q3d × 6), in combination with paclitaxel (15 mg/kg, i.p., q3d × 6), significantly inhibited the growth of ABCC10-expressing tumors in athymic nude mice. NVP-BHG712 administration significantly increased the levels of paclitaxel in the tumors but not in plasma compared to paclitaxel alone. The combination of NVP-BHG712 and paclitaxel could serve as a novel and useful therapeutic strategy to attenuate paclitaxel resistance mediated by the expression of the ABCC10 transporter.

## INTRODUCTION

Paclitaxel is a clinically used chemotherapeutic drug, but its use can elicit resistance to various anticancer drugs in certain types of cancers [1]. The efficacy of paclitaxel can be attenuated by the overexpression of multidrug efflux transporters [2-4], altered metabolism, decreased sensitivity to apoptosis [5, 6], alterations in microtubule dynamics, diminished interaction of paclitaxel to its cellular target [7, 8] and genetic polymorphisms [9]. These aforementioned mechanisms of resistance typically produce chemotherapeutic failure. Recent data indicate that paclitaxel resistance occurs via its active efflux from cancer cells due to the expression of the ATP-binding cassette subfamily C member 10 (ABCC10), also known as multidrug resistant protein 7 (MRP7) [1, 3]. The human *ABCC10* gene is located on chromosome 6p21.1. ABCC10 is a 171-kDa protein that contains three membrane-spanning domains (MSDs) and two nucleotide-binding domains (NBDs) [1, 10]. ABCC10 also mediates the cellular efflux of several other antineoplastic drugs, including docetaxel, vincristine, vinblastine, vinorelbine, cytarabine, gemcitabine, 2′,3′-dideoxycytidine, 9-(2-phosphonyl methoxyethyl)adenine (PMEA), and epothilone B, and endogenous substances such as estradiol-17β-D-glucuronide (E217βG) and leukotriene C4 [3]. The expression of the ABCC10 transporter is positively correlated with paclitaxel resistance in non-small cell lung cancer (NSCLC) [1, 11, 12]. The *ABCC10* transcript is expressed (in order of highest to lowest) in the pancreas, liver, placenta, lungs, kidneys, brain, ovaries, lymph nodes, spleen, heart, leukocytes, and colon [13]. Another group reported that *ABCC10* mRNA is highly expressed in various tissues, including the kidneys, brain, and colon, suggesting that it is involved in the transport of drugs and other endogenous molecules [14].

NVP-BHG712 (4-methyl-3-(1-methyl-6-(pyridin-3-yl)-1H-pyrazolo[3,4-d]pyrimidin-4-ylamino)-N-(3-(trifluoromethyl)phenyl)benzamide) is a specific EphB4 receptor (receptor cloned from erythropoietin-producing hepatocellular carcinoma) inhibitor that blocks vascular endothelial growth factor mediated angiogenesis *in vivo* [15]. Previous preclinical studies from our lab have shown that the ABCC10 transporter efflux function is inhibited by tyrosine kinase inhibitors, such as nilotinib and masitinib [1, 16-18]. For the first time, we report that *in vitro*, NVP-BHG712 significantly inhibits the efflux activity of the ABCC10 in HEK293 cells overexpressing the ABCC10 transporter. Furthermore, we demonstrate that NVP-BHG712 enhanced paclitaxel-mediated inhibition of the growth of ABCC10-expressing tumor in a tumor xenograft mouse model *in vivo* and report the tumor and plasma concentration of paclitaxel in mice administered with paclitaxel and NVP-BHG712.

## RESULTS

### NVP-BHG712 significantly enhances the sensitivity of HEK293/ABCC10 cells to paclitaxel

**Figure 1:**
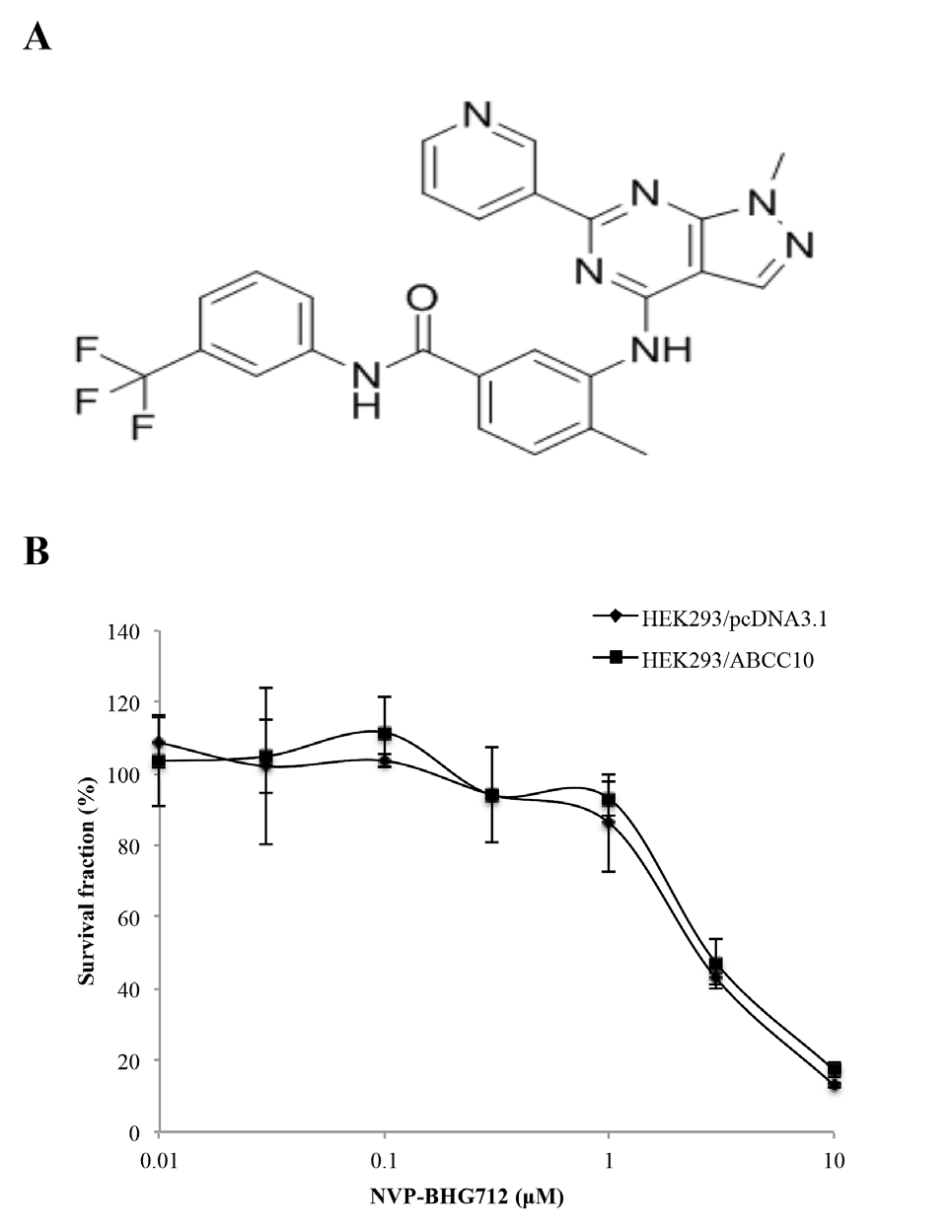
Cytotoxicity of NVP-BHG712 A, the chemical structure of NVP-BHG712, 4-Methyl-3-[[1-methyl-6-(3-pyridinyl)-1H-pyrazolo[3,4-d]pyrimidin-4-yl]amino]-N-[3-(trifluoromethyl)phenyl]benzamide. B, cytotoxicity of NVP-BHG712 was determined by the MTT assay in HEK293/pcDNA3.1 and HEK/ABCC10 cells. Error bars indicate SD.

Before determining the effect of NVP-BHG712 on paclitaxel resistance, we examined its effect on the growth of the cell lines used in our study. Based on the cytotoxicity assay, we chose to use NVP-BHG712 (Fig. 1A and B) at concentrations of 0.25 μM and 0.5 μM because at these concentrations, at least 80-90% of the cells survived. NVP-BHG712, at 0.25 μM and 0.5 μM, significantly decreased the resistance to paclitaxel in the HEK293/ABCC10 cell line as compared to the control HEK293/pcDNA3.1 cells (Table 1). Cepharanthine (2.5 μM), which has been shown to inhibit ABCC10 function, significantly decreased the resistance of HEK293/ABCC10 to paclitaxel as compared to the parental HEK293/pcDNA3.1 cells [19]. The incubation of cells with 0.5 μM of NVP-BHG712 or 2.5 μM cepharanthine did not significantly alter the IC_50_ values of cisplatin, which is not a substrate for ABCC10 in HEK293/pcDNA3.1 and HEK293/ABCC10 cells (Table 1) [20]. NVP-BHG712 also significantly increased the response of HEK293/ABCC10 cells to docetaxel and vinblastine, which are substrates of ABCC10 (Supplemental Table 1). In order to determine the effect of NVP-BHG712 on the ABCB1, ABCC1 and ABCG2 transporters, we used the HEK293/ABCB1, HEK293/ABCC1, and ABCG2-482-R2, ABCG2-482-G2 and ABCG2-482-T7 cells, which express the ABCB1, ABCC1 and ABCG2 transporters, respectively. We used paclitaxel, vinblastine and colchicine as substrates for ABCB1, vincristine for ABCC1 and mitoxantrone for ABCG2. NVP-BHG712 partially reversed ABCB1-, ABCC1- and ABCG2-mediated drug resistance (Supplemental Table 2, 3 and 4).

**Table:** 

Compounds	HEK293/pcDNA3.1		HEK293/ABCC10	
	IC_50_ ± SD^a^ (nM)	FR^b^	IC_50_ ± SD (nM)	FR
Paclitaxel	10.60 ± 1.3	[1.0]	100.39 ± 6.4	[9.4]
+NVP-BHG712 0.25 μM	10.20 ± 0.9	[1.0]	20.80 ± 5.8^**^	[2.0]
+NVP-BHG712 0.5 μM	9.60 ± 0.5	[0.9]	10.17 ± 1.0^**^	[1.0]
+Cepharanthine 2.5 μM	9.72 ± 0.7	[0.9]	10.29 ± 0.6^**^	[1.0]
Cisplatin	1629.91 ± 173.8	[1.0]	1522.56 ± 145.9	[0.9]
+NVP-BHG712 0.5 μM	1381.91 ± 226.9	[0.8]	1351.02 ± 102.9	[0.8]
+Cepharanthine 2.5 μM	1818.97 ± 103.60	[1.1]	1995.65 ± 186.3	[1.2]

### NVP-BHG712 significantly potentiates the anticancer activity of paclitaxel in an ABCC10-expressing tumor-xenograft model

**Figure 2:**
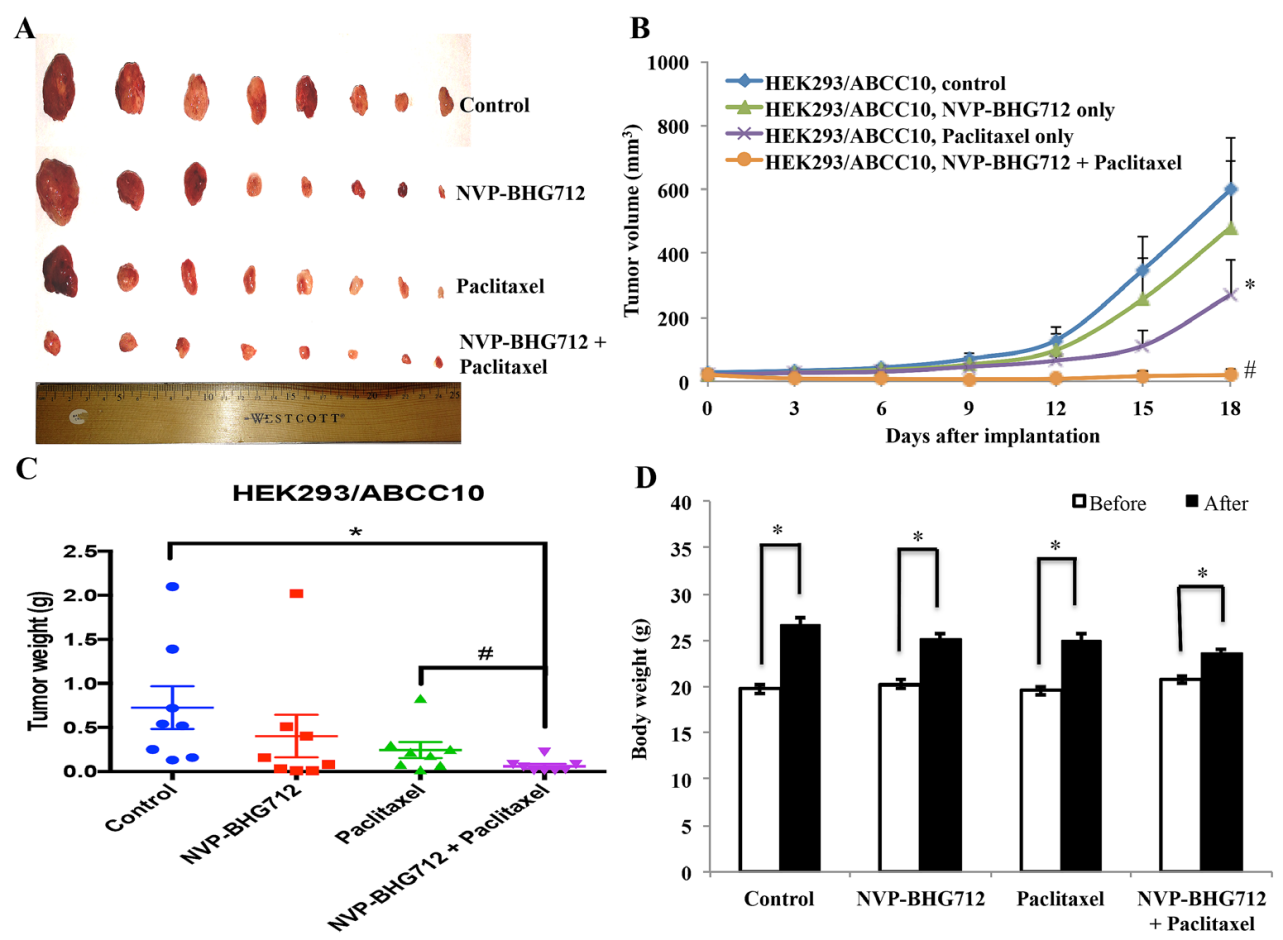
The effect of NVP-BHG712 on the growth of ABCC10-expressing tumors in athymic nude mice A, images of excised HEK293/ABCC10 tumors implanted subcutaneously in athymic NCR nude mice (n = 8) that were treated with vehicle, paclitaxel, NVP-BHG712 or combination of NVP-BHG712 plus paclitaxel, at the end of the 18-day treatment period. Results are representative of 3 independent experiments performed. B, the changes in tumor volume over time following implantation. Data points represent mean tumor volume for each treatment group (n = 8). Error bars represent SEM. *: *p* < 0.05 versus the vehicle group; #: *p* < 0.01 versus paclitaxel alone group. C, mean weight (n = 8) of the excised HEK293/ABCC10 tumors from the mice treated with with vehicle, paclitaxel, NVP-BHG712 or combination of NVP-BHG712 plus paclitaxel, at the end of the 18-day treatment period. Error bars represent SEM. *: *p* < 0.05 versus vehicle group; #: *p* < 0.05 versus the paclitaxel group. D, the changes in mean body weight of mice (n = 8) before and after treatment. NS: not statistically significant (*p* > 0.05).

The i.p. dose of paclitaxel (15 mg/kg) used in this study was determined after a series of pilot experiments which indicated that it produced significant resistance in HEK293/ABCC10 tumor-xenograft model compared to the HEK293/pcDNA3.1 tumor-xenograft model. NVP-BHG712, alone (25 mg/kg p.o.) or in combination with paclitaxel, did not produce any visible toxicity or phenotypic changes in the male athymic NCR nude mice. The tumors expressing the ABCC10 transporter showed significant resistance to the 15 mg/kg i.p. dose of paclitaxel (Fig. 2A, B and C). This is in contrast to the tumors composed of HEK293/pcDNA3.1 cells, which were almost completely eliminated by 15 mg/kg of paclitaxel (Supplemental Fig. 1A and B). No apparent weight loss was observed among the treatment groups compared to animals treated with vehicle (Fig. 2D). The intergroup comparisons of the body weights are shown in Supplemental Fig. 2. NVP-BHG712 (25 mg/kg, p.o.), in combination with paclitaxel (15 mg/kg, i.p.), significantly decreased the sizes, weights and volumes of the tumors expressing the ABCC10 transporter (HEK293/ABCC10) over a period of 18 days, compared to animals treated with vehicle, NVP-BHG712 alone or paclitaxel alone (*p* < 0.05; Fig. 2A, B, C and D, respectively). These results suggest that NVP-BHG712 significantly attenuates paclitaxel resistance in tumors expressing the ABCC10 transporter.

### NVP-BHG712 significantly increases paclitaxel levels in the tumor but not in the plasma in the mice expressing ABCC10 transporter

**Figure 3:**
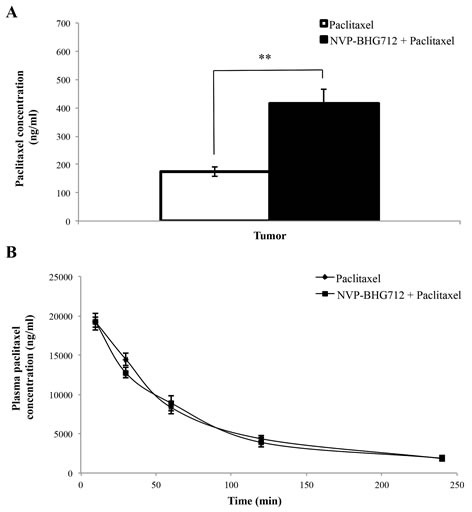
The effect of NVP-BHG712 and paclitaxel co-administration on plasma and intratumoral paclitaxel concentrations in mice A, paclitaxel concentrations in HEK293/ABCC10 tumors (n = 7) after 240 min following administration and B, plasma at 10, 30, 60, 120, 240 min following administration (n = 7). Columns and error bars represent mean ± SEM. **: *p* < 0.01 versus paclitaxel only group.

In a separate study, we measured the plasma and tumor concentrations of paclitaxel in animals pretreated with vehicle or NVP-BHG712 (25 mg/kg, p.o.) prior to the administration of paclitaxel (15 mg/kg, i.v.). The pharmacokinetic data showed that co-administration of NVP-BHG712 and paclitaxel significantly increased the intratumoral concentration of paclitaxel (434.58 ± 124.49 ng/ml) as compared to paclitaxel administration alone (160.13 ± 41.12 ng/ml, *p* < 0.01) after 240 min following administration (Fig. 3A). The combination of NVP-BHG712 and paclitaxel did not affect plasma levels of paclitaxel (Fig. 3B) after 240 min following administration. These data suggest that NVP-BHG712-induced increase in the efficacy of paclitaxel in tumors expressing the ABCC10 is due at least in part to its direct inhibition of the transporter activity of ABCC10, thereby increasing the intracellular accumulation of paclitaxel.

### A comparison of the effect of NVP-BHG712 and TKIs on the efficacy of paclitaxel

**Figure 4:**
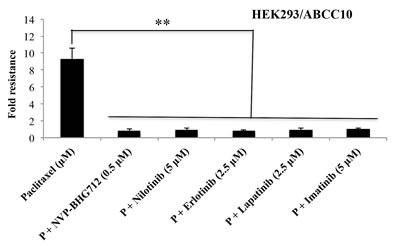
Sensatization to paclitaxel with or without TKIs The sensitization effect of TKIs in combination with paclitaxel is shown in ABCC10-expressing HEK293/ABCC10 cells. The data points represent the mean ± SD of at least three independent experiments performed in triplicate.**: *P* < 0.01 versus the control group.

Previously, we have reported that the efflux activity of the ABCC10 transporter is significantly inhibited by various TKIs [3]. Therefore, we compared the efficacy of NVP-BHG712 to that of the TKIs nilotinib, erlotinib, lapatinib and imatinib [3, 21, 22]. In HEK293/ABCC10 cells overexpressing the ABCC10 transporter, NVP-BHG712 was significantly potent as comparable to the TKIs in potentiating the efficacy of paclitaxel (Fig. 4). The concentration-response curves of NVP-BHG712 and other inhibitors in combination with paclitaxel are shown in Supplemental Fig. 3.

### NVP-BHG712 significantly increases the cellular accumulation of [^3^H]-paclitaxel in HEK293/ABCC10 cells

**Figure 5:**
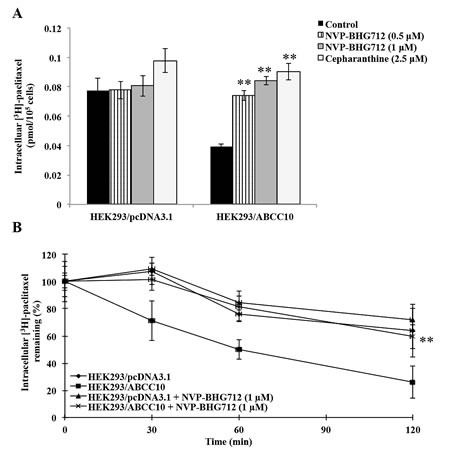
NVP-BHG712 increases the cellular accumulation of [H]-paclitaxel and decreases the cellular efflux of [H]-paclitaxel in HEK293/ABCC10 cells A, the accumulation of [^3^H]-paclitaxel was measured after the cells (HEK293/pcDNA3.1 and HEK293/ABCC10) were pre-incubated with or without NVP-BHG712 or cepharanthine for 2 h at 37°C and then incubated with 0.01 μM [^3^H]-paclitaxel for another 2 h at 37°C. Error bars indicate SD. **: *p* < 0.01 versus the control group. B, a time course versus the percentage of intracellular [^3^H]-paclitaxel remaining (%) was plotted (0, 30, 60, 120 min). Error bars indicate SD. **: *p* < 0.01 versus the control group.

In order to determine the effect of NVP-BHG712 on the function of the ABCC10 transporter, we examined the effect of NVP-BHG712 on the intracellular accumulation of [^3^H]-paclitaxel in HEK293/ABCC10 cells. NVP-BHG712 produced a significant, concentration-dependent increase in the intracellular accumulation of [^3^H]-paclitaxel compared to cells incubated with the solvent buffer for NVP-BHG712 (Fig. 5A). In addition, the results obtained with NVP-BHG712 were comparable to those obtained with the known ABCC10 transport inhibitor cepharanthine.

### NVP-BHG712 significantly decreases the cellular efflux of [^3^H]-paclitaxel in HEK293/ABCC10 cells

We further determined the amount of [^3^H]-paclitaxel present in the cells following incubation with NVP-BHG712. The amount of [^3^H]-paclitaxel present in the intracellular lysate of HEK293/ABCC10 cells was significantly lower compared to that of HEK293/pcDNA3.1 cells due to active efflux of the [^3^H]-paclitaxel via ABCC10. However, over a period of time (0, 30, 60, 120 min) NVP-BHG712 (1 μM) significantly reduced the efflux of [^3^H]-paclitaxel in HEK293/ABCC10 cells (Fig. 5B).

### NVP-BHG712 has no effect on the expression levels of ABCC10 and EphB4

**Figure 6:**
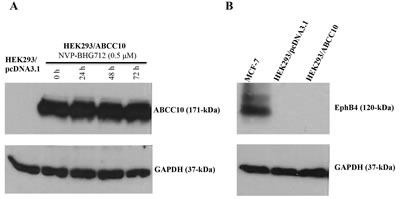
Immunoblot analysis of the expression of ABCC10 transporter and EphB4 A, the expression of the ABCC10 protein in HEK293/ABCC10 cells treated with NVP-BHG712 (0.5 μM) for the indicated period of time, determined by immunoblot analysis. GAPDH was used as a loading control. B, the expression of EphB4 in MCF-7, HEK293/pcDNA3.1 and HEK293/ABCC10 cells. Data presented are representative of 3 independent experiments.

Immunoblot analysis for the ABCC10 protein indicated a band with a molecular weight of about 171-kDa in the HEK293/ABCC10 cell lysates, consistent with the presence of the ABCC10 protein. In contrast, this band was not present in HEK293/pcDNA3.1, indicating the absence of the ABCC10 protein (Fig. 6A). In order to confirm NVP-BHG712's reversal action was not due to a decrease in the expression of the ABCC10 protein, we incubated the cells with NVP-BHG712 (0.5 μM) for 0, 24, 48 and 72 h. NVP-BHG712 did not significantly alter the expression levels of the ABCC10 transporter in the HEK293/ABCC10 cells (Fig. 6A). In a separate set of experiment, we found that the EphB4 (120-kDa) protein (that is specific target of NVP-BHG712) was not detected in HEK293/pcDNA3.1 or HEK293/ABCC10 cell lysates where MCF-7 cell lysate was used as positive control for EphB4 protein expression (Fig. 6B). These findings suggested that the reversal of paclitaxel resistance by NVP-BHG712 did not result from its alteration in ABCC10 or EphB4 protein expression.

## DISCUSSION

Our preclinical study indicated that NVP-BHG712, in combination with paclitaxel, significantly attenuated tumor growth in athymic nude mice implanted with HEK293 cells expressing the ABCC10 transporter in a tumor-xenograft model (Fig. 2A, B and C). NVP-BHG712 essentially restored the sensitivity of tumors expressing ABCC10 to paclitaxel any phenotypic change. Furthermore, the pharmacokinetic data indicated that NVP-BHG712 significantly increased the levels of paclitaxel in ABCC10-expressing tumors in mice but did not increase the plasma paclitaxel levels compared to paclitaxel alone.

One of the major findings of this study was that NVP-BHG712 significantly enhanced the sensitivity of HEK293/ABCC10 cells to paclitaxel and to other substrates, such as docetaxel and vinblastine. In addition, NVP-BHG712 did not significantly increase the toxic effect of cisplatin, a drug that is not a substrate for the ABCC10 transporter, further supporting the specificity of NVP-BHG712. To our knowledge, this is the first study demonstrating that NVP-BHG712 potentiates the cytotoxic effects of paclitaxel in cells expressing ABCC10 transporter. Apart from ABCC10, NVP-BHG712 partially alters the IC_50_ values of anticancer drugs in HEK293/ABCB1, HEK293/ABCC1, and ABCG2-482-R2, ABCG2-482-T7 and ABCG2-482-G2 cell lines, respectively [23, 24], although not to the magnitude seen with ABCC10. These results suggest that NVP-BHG712 strongly potentiates ABCC10-mediated paclitaxel resistance and moderately potentiates ABCB1, ABCC1 and ABCG2-mediated drug resistance.

Our results also indicated that NVP-BHG712 (0.5 or 1 μM) produced a significant concentration-dependent increase in the intracellular accumulation of [^3^H]-paclitaxel in HEK293 cells that expressed the ABCC10 transporter. In addition, consistent with the aforementioned results, NVP-BHG712 (1 μM) significantly decreased the efflux of [^3^H]-paclitaxel from cells expressing the ABCC10 transporter. These findings tentatively suggest that NVP-BHG712 increases the sensitivity of ABCC10-expressing cells to paclitaxel by inhibiting its efflux from the cells. This could be due the direct interaction of NVP-BHG712 with the ABCC10 transporter, although this remains to be proven. NVP-BHG712 (0.5 μM) did not significantly alter the expression of the ABCC10 protein (171-kDa protein in the immunoblot assay in HEK293/ABCC10 cells, Fig. 6). This finding suggests that NVP-BHG712 re-sensitizes HEK293/ABCC10 cells to paclitaxel without significantly altering its expression.

The current study did not determine the effect of NVP-BHG712 on cancer cells. Nonetheless, several studies suggest that the overexpression of ABCC10 in cancer cells produces resistance to specific antineoplastic drugs. For example, there is a significant negative correlation between ABCC10 overexpression and paclitaxel sensitivity in human non-small lung cancer cells [11]. Similarly, Bessho et al. (2009) reported that the overexpression of ABCC10 is significantly correlated with venorebline resistance in non-small cell lung cancer [25]. The expression of the ABCC10 produces resistance to docetaxel in mouse salivary gland adenocarcinoma cell lines [26]. Interestingly, mouse embryo fibroblasts that do not express ABCC10 are highly sensitive to the *in vivo* toxic effects of paclitaxel [27]. Thus, future studies should be conducted to determine the effect of NVP-BHG712 on cancer cells that have obtained the acquired resistance phenotype due to overexpression of the ABCC10 transporter.

Our *in vivo* and *in vitro* data indicate that NVP-BHG712 surmounts paclitaxel resistance mediated by the overexpression of the ABCC10 transporter. Furthermore, *in vitro*, NVP-BHG712 significantly (but not completely) reverses multi-drug resistance mediated by the overexpression of the ABCB1, ABCC1 and ABCG2 transporters. These data, provided they could be extrapolated to humans, suggest that NVP-BHG712 may be used to overcome resistance mediated by the overexpression of multiple ABC transporters in cancer cells. In this study, the plasma concentration of paclitaxel in the xenografted mice was approximately 2000 ng/ml for 4 h after the administration of 15 mg/kg of paclitaxel. In addition, the plasma levels of paclitaxel in our study were not significantly altered by the administration of 25 mg/kg i.p. of NVP-BHG712. Thus, it is unlikely that the systemic administration of NVP-BHG712 would potentiate the toxic effects of paclitaxel. However, NVP-BHG712 significantly blocks the efflux of paclitaxel from cells overexpressing specific ABC transporters, and this potentiates the efficacy of paclitaxel. To our knowledge, NVP-BHG712 is the only inhibitor of the ABCC10 transporter that does not significantly alter the plasma levels of paclitaxel in athymic nude tumor xenograft mice overexpressing the ABCC10 transporter. Moreover, NVP-BHG712 is the most potent inhibitor of ABCC10 (0.5 μM) discovered so far that has favorable *in vivo* properties.

Finally, it should be noted that NVP-BHG712 is a potent antagonist of EphB4 receptor [15]. Thus it could be argued that NVP-BHG712 modulates paclitaxel resistance as a result of its effect on EphB4 or proteins downstream of EphB4 or ABCC10. However, this possibility could be ruled out, as the cell lines used in this study did not express EphB4 (Fig. 6B). Thus, sensitization to paclitaxel by NVP-BHG712 is only because of its action on the ABCC10 transporter.

Collectively, our results show that NVP-BHG712, a receptor tyrosine kinase inhibitor, has previously unknown function in sensitizing ABCC10-expressing cells to the ABCC10 substrate paclitaxel *in vitro* and *in vivo*. This effect was likely due in part to a blockade of paclitaxel efflux by ABCC10 as opposed to the alteration of the expression of the ABCC10 protein. In addition, NVP-BHG712 potentiates the anti-tumor efficacy of paclitaxel *in vivo* while exhibiting excellent pharmacokinetic profile. Thus, the combination therapy of NVP-BHG712 and paclitaxel could represent an effective strategy to treat patients with cancers that are resistant to paclitaxel as a result of the expression of the ABCC10 transporter.

## MATERIALS AND METHODS

[^3^H]-paclitaxel (37.9 Ci/mmol) was purchased from Moravek Biochemicals, Inc. (Brea, CA). Dulbecco modified Eagle medium (DMEM), fetal bovine serum (FBS), phosphate buffer saline (PBS), 10,000 IU/ml penicillin and 10,000 μg/ml streptomycin, and trypsin 0.25% were purchased from Hyclone (Waltham, MA). Monoclonal antibody against GAPDH was purchased from Cell Signaling Technologies (Beverly, MA). The polyclonal antibody PA5-23652 for ABCC10 was obtained from Thermo scientific (Rockford, IL). Monoclonal antibody 3D7G8 against EphB4 was obtained from Life technologies, Invitrogen (New York, NY). NVP-BHG712 was a gift from Novartis (Basel, Switzerland). Cepharanthine was generously donated by Kakenshoyaku Co. (Tokyo, Japan). PAK-104P was a gift from Nissan Chemical Industries (Tokyo, Japan). Paclitaxel, docetaxel, colchicine, mitoxantrone, vincristine, vinblastine and cisplatin were purchased from Tocris Bioscience (Ellisville, MO). 3-(4, 5-Dimethylthiazol-yl)-2,5-diphenyltetrazolium bromide (MTT), Dimethyl sulfoxide (DMSO) and verapamil were obtained from Sigma-Aldrich Co. (St. Louis, MO).

### Cell lines and cell culture

The parental empty pcDNA3.1 plasmid-transfected cell line, HEK293/pcDNA3.1, and the cell line stably transfected with pcDNA3.1 containing an expression construct encoding ABCC10 (HEK293/ABCC10) were used in all experiments as previously reported [1]. The HEK293/ABCB1 and HEK293/ABCC1 cells were kindly provided by Dr. Suresh V. Ambudkar (NCI, NIH, MD) in 2012. The ABCG2-482-R2, ABCG2-482-G2 and ABCG2-482-T7 cells were kindly provided by Dr. Susan E. Bates (NCI, NIH, MD) in 2012. MCF-7 cell line was obtained from Ray Biotech, Inc. (Norcross, GA). The HEK293/pcDNA3.1, HEK293/ABCC10, HEK293/ABCB1, HEK293/ABCC1, ABCG2-482-R2, ABCG2-482-G2, ABCG2-482-T7 and MCF-7 cell lines were cultured in DMEM, supplemented with 10% heat-inactivated FBS and 1% of 100 times diluted 10,000 IU/ml penicillin-10,000 μg/ml streptomycin [28]. All *in vitro* experiments were conducted at 60% to 80% cell confluency. Cells used in the *in vivo* experiments were trypsinized and centrifuged at 2000 rpm for 2 min at 25°C, washed twice with PBS, and reconstituted in DMEM at a concentration of 1×10^7^ cells. HEK293/pcDNA3.1 and HEK293/ABCC10 cell lines were authenticated using short tandem repeat analysis by the American Type Culture Collection (Manassas, VA). The HEK293/ABCB1, HEK293/ABCC1, ABCG2-482-R2. ABCG2-482-G2, ABCG2-482-T7 and MCF-7 cell lines were not authenticated.

### Cell viability assay

A modified MTT assay was performed to detect the sensitivity of the cells to the antineoplastic drugs *in vitro* [29-31]. Approximately 5000 HEK293/pcDNA3.1 and HEK293/ABCC10 cells were placed in each well. Every MTT assay was run in triplicate and drugs tested included paclitaxel (0.001 to 1 μM), docetaxel (0.001 to 1 μM), vinblastine (0.001 to 1 μM), vincristine (0.001 to 1 μM), cisplatin (0.1 to 100 μM), NVP-BHG712 (0.25 μM and 0.5 μM), cepharanthine (2.5 μM), verapamil (2.5 μM), and PAK-104P (5 μM). After seeding cells in 180 μl of medium in 96-well plates and incubating for 24 h at 37°C, 20 μl of the appropriate antineoplastic drug at various concentrations was added (20 μl of fixed concentration of test compound for reversal agent were added 1 h prior to adding anticancer drugs). Subsequently, the antineoplastic drugs, in DMEM supplemented with 10% FBS, were incubated at 37°C for 72 h. After 72 h, 20 μl MTT (4 mg/ml) was added to each well. The plates were incubated at 37°C for another 4 h. The MTT with medium was removed from each well, and 100 μl of DMSO was added to each well. The absorbance was measured at 570 nm by an Opsys microplate reader (Dynex Technologies, VA). The degree of resistance was calculated by dividing the IC_50_ (calculated using Bliss method) for resistant cells by that of the parental sensitive cells [32]. The degree of the reversal of the resistance was calculated by dividing the IC_50_ for cells with the anticancer drug in the absence of NVP-BHG712 or other reversal compounds by that obtained in the presence of NVP-BHG712 or cepharanthine.

### Animal preclinical efficacy trail design

Male athymic NCR (nu/nu) nude mice (NCRNU M, homozygous, albino; 18-25 g; 4-6 week; Taconic Farms, NY) were used for the tumor-xenograft experiments. All animals were maintained on an alternating 12 h light/dark cycle with *ad libitum* access to water and rodent chow. The ABCC10-expressing HEK293/ABCC10 model was designed for the first time and used as previously described by Chen and colleagues [1, 16, 33-35]. Briefly, HEK293/pcDNA3.1 and HEK/ABCC10 (1.0 × 10^7^) cells were injected s.c. (0.2 ml) under the armpits. When the tumors reached a mean diameter of 0.5 cm (day 0), the mice were randomized into four groups (n = 8) and treated with either (a) vehicle (10% N-methyl-pyrrolidinone, 90% polyethylene glycol 300) (q3d x 6), (b) paclitaxel (15 mg/kg, i.p., q3d × 6), (c) NVP-BHG712 diluted in 10% N-methyl-pyrrolidinone, 90% polyethylene glycol 300 (25 mg/kg, p.o., q3d × 6), or (d) NVP-BHG712 (25 mg/kg, p.o., q3d × 6, given 1 h before giving paclitaxel) + paclitaxel (15 mg/kg, i.p., q3d × 6). Paclitaxel was prepared by dissolving 6 mg paclitaxel in 50% dehydrated alcohol (EMD, MA) and 50% Cremophor ELP (BASF, NJ). The tumor sizes were measured using calipers and body weights were recorded [32]. The body weight of the animals was monitored every 3^rd^ day to adjust the drug dosage and to determine treatment-related toxicities as well as tumor progression. The two perpendicular diameters of tumors were recorded every 3^rd^ day and tumor volume was estimated, as described previously [32, 33]. All the animals were killed by terminal bleeding through cardiac puncture under isofluorane anesthesia, and plasma was collected and tumor tissue was excised and stored at −80°C [1, 36]. All mice were maintained at the St. John's University Animal Facility. The IACUC at St. John's University approved this project, and the research was conducted in compliance with the Animal Welfare Act and other federal statutes. Animals were treated humanely and cared for in accordance with guidelines set forth by the American Association for Accreditation of Laboratory Animal Care and the US Public Health Service *Policy on Humane Care and Use of Laboratory Animals*, and all studies were approved and supervised by the IACUC at St. John's University.

### Collection of plasma and tissues

In separate experiments, mice bearing HEK293/ABCC10 tumors were divided into two groups: 1) vehicle pretreatment (given 1 h before paclitaxel 15 mg/kg); 2) NVP-BHG712 pretreatment (25 mg/kg p.o.) and paclitaxel 15 mg/kg (n = 7). After treatment, animals were anesthetized (using isofluorane) and blood (50 μl) was obtained using supraorbital puncture and placed in heparinized tubes and plasma was harvested at 10, 30, 60, 120 or 240 min after paclitaxel administration in both groups. In addition, the tumors were removed, weighed, snap frozen in liquid nitrogen, and stored at −80°C until analysis [16].

### Extraction of paclitaxel from plasma and tissue homogenate samples

A simple, one-step protein precipitation with acetonitrile was used for sample preparation. Tumor and lung tissues were homogenized in saline (1:2 (v/v)). Paclitaxel was extracted from plasma and tissue homogenate samples by precipitation with acetonitrile in 1:1 and 1:2 ratios (v/v), respectively. Samples were vortexed for 1 min, followed by centrifugation for 10 min at 10,000 rpm. The supernatant was transferred to insert vials from which 20 μl was injected onto the HPLC column. Samples with concentrations higher than the calibration range limit were appropriately diluted to fit within the working calibration curve.

### Quantification of paclitaxel

The quantification of paclitaxel in plasma and tumors was conducted using an isocratic Shimadzu LC-20AB HPLC equipped with an Shimadzu SIL-20A HT autosampler and LC-20AB pump connected to a Dgu-20A3 degasser (Shimadzu, OR), according to the method described by Gill et al. [37]. A reverse-phase, Phenomenex Luna C18 column (250 × 4.6 mm i.d., 5 μm; Phenomenex, CA) with an ODS guard column (4 mm × 3 mm; Phenomenex, CA), was used. The injection volume was 20 μl, and the mobile phase used for the separation of paclitaxel in plasma and tissue homogenate samples consisted of acetonitrile and water (53:47, v/v) delivered at a flow rate of 1.0 ml/min. For paclitaxel detection, the Shimadzu UV SPD-20A (Shimadzu, OR) detector set was at 227 nm. Data acquisition and analysis was achieved using LC Solution software version 1.22 SP1 (Shimadzu, OR). All samples were analyzed in duplicate. Under these chromatographic conditions, the total run time was 15 min with a retention time of 12 min for paclitaxel. Standard curves for paclitaxel in plasma and tissue homogenates were prepared in the ranges of 25-5000 ng/ml. The analytical method described in this work has been already established and validated previously [16, 37].

### [^3^H]-paclitaxel accumulation assay

The HEK293/pcDNA3.1 and HEK293/ABCC10 cell lines were harvested at 80% confluency. All cell lines were trypsinized and cell count was done using a hemocytometer. Approximately 7 × 10^6^ cells were incubated at 37°C in DMEM, supplemented with 10% FBS with and without NVP-BHG712 (0.5 or 1 μM) for 2 h. Subsequently, HEK293/pcDNA3.1 and HEK293/ABCC10 cells were incubated with 0.01 μM [^3^H]-paclitaxel in presence or absence of NVP-BHG712 (0.5 or 1 μM) for 2 h. Following incubation, the medium was removed and the cells were rinsed three times with cold PBS. The cells were lysed using 200 μl of lysis buffer (pH 7.4; containing 1% Triton X-100 and 0.2% SDS) and transferred to scintillation vials. Each sample was placed in scintillation fluid (5 ml) and radioactivity was measured using a liquid scintillation counter (Packard Instrument, IL) [38-40].

### [^3^H]-paclitaxel efflux assay

Cells were exposed to the same procedure as stated in the drug accumulation experiment and then incubated in fresh medium at 37°C at various times (0, 30, 60, and 120 min) in the presence or absence of the reversal agent (NVP-BHG712 at 1 μM). After washing three times with ice-cold PBS, the cells were lysed using 200 μl lysis buffer and transferred to scintillation vials. Each sample was placed in scintillation fluid and radioactivity was measured in a liquid scintillation counter (Packard Instrument, IL) [41, 42].

### Immunoblot analysis

In order to determine if NVP-BHG712 1) affects the expression of ABCC10 and 2) blocks EphB4, cells were incubated with NVP-BHG712 at 0.5 μM for different time periods (0, 24, 48, and 72 h). Then, approximately 6 × 10^5^ cells were harvested and suspended in PBS, centrifuged at 2000 rpm for 2 min, followed by two washings with PBS. The lysis buffer (1X PBS, 0.1% SDS, 1% Nonidet P-40, 0.5% sodium deoxycholate, and 100 mg/ml p-aminophenylmethylsulfonyl fluoride) and 1% aprotinin was added to the suspension followed by vortexing. The resuspended cells were kept on ice for 30 min, followed by centrifugation at 12,000 rpm for 20 min. The supernatant was separated and stored in −80°C for the experiment. Protein concentrations in the lysates were determined by the bicinchonic acid based protein assay. Equal amounts of total cell lysates (40 μg protein) were resolved by SDS-PAGE electrophoresis and electrophoretically transferred onto PVDF membranes. The membranes were incubated in a blocking solution in TBST buffer (10 mM Tris-HCl, pH 8.0, 150 mM NaCl, and 0.1 % Tween 20) for 1 h at room temperature. Subsequently, the membranes were immunoblotted overnight with primary monoclonal antibodies against ABCC10 at 1:1000 dilution, EphB4 or GAPDH at 1:500 or 1:1000 dilution respectively, at 4°C and were then incubated for 3 h at room temperature with HRP-conjugated secondary antibody (1:1000 dilution). The protein-antibody complex was detected using chemiluminescence [41-43].

### Statistical analyses

All experiments were repeated at least three times and the differences were determined using the two-tailed student's *t*-test and statistical significance was determined at *p* < 0.05.

## SUPPLEMENTARY MATERIAL, TABLES AND FIGURES


