# Supplementary material for: The small molecule tyrosine kinase inhibitor NVP-BHG712 antagonizes ABCC10-mediated paclitaxel resistance: a preclinical and pharmacokinetic study
Source: Oncotarget. 2014 Oct 28;6(1):510–21. doi: 10.18632/oncotarget.2638 (PMC4381611; doi:10.18632/oncotarget.2638)
Supplement: Supplementary file 1 [file oncotarget-06-510-s001.pdf]

The small molecule tyrosine kinase inhibitor NVP-BHG712 antagonizes ABCC10-mediated paclitaxel resistance: a preclinical and pharmacokinetic study

Supplementary Material

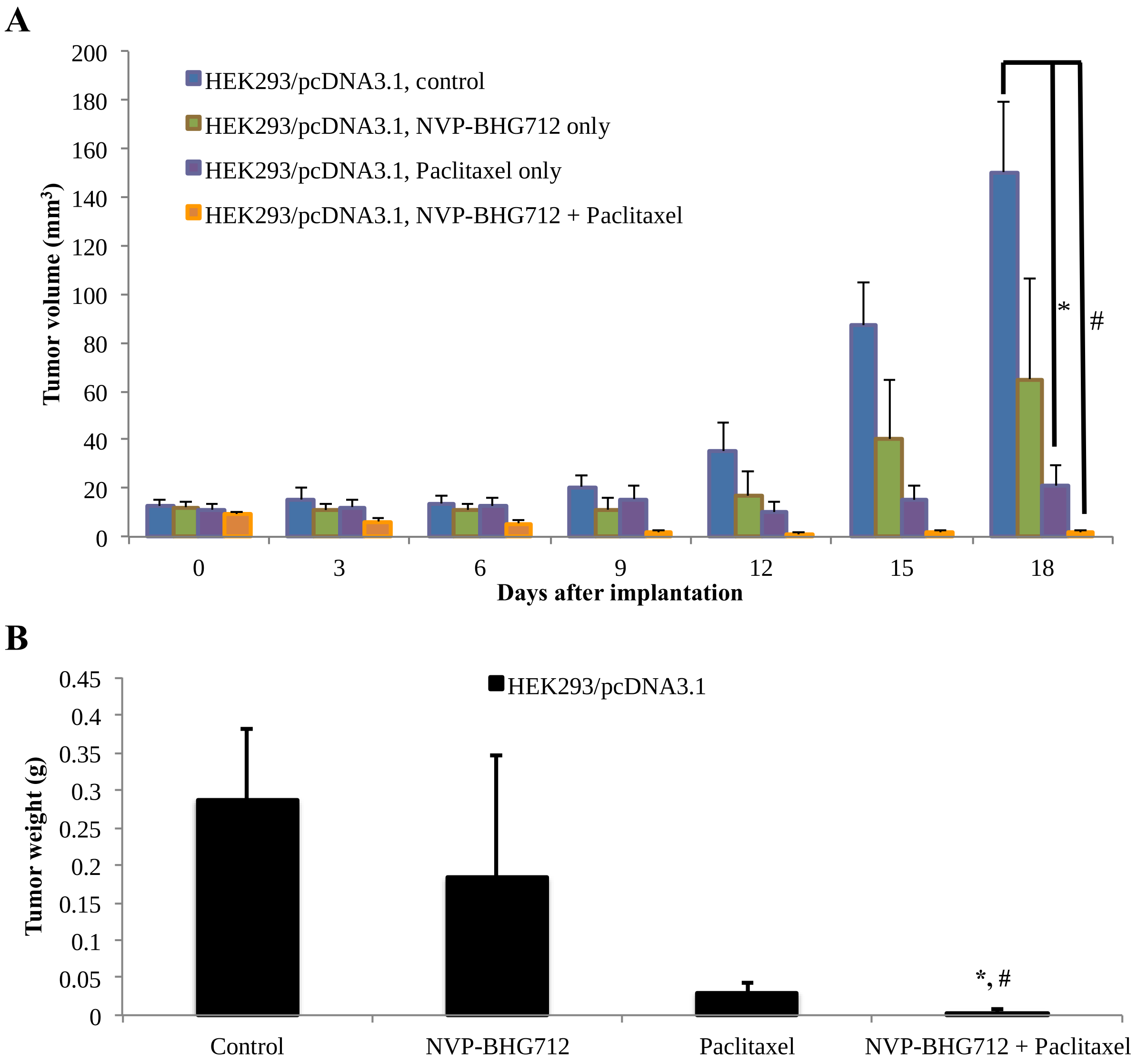

Supplemental Figure 1: The effect of NVP-BHG712 administration on HEK293/pcDNA3.1 tumor growth *in vivo*. (A) Changes in tumor volume over time are shown. Bar graphs represent

mean tumor volume for each experimental group (n = 8) during treatment. Each point on the graph represents the mean tumor volume (mm^3) at a particular time after treatment. Error bars represent SEM. *, $p < 0.05$ versus the vehicle group. **(B)** The bar graph represents the mean tumor weight (n = 8) of the excised HEK293/pcDNA3.1 tumors from different mice. The treatments were as follows: vehicle, paclitaxel, NVP-BHG712 and NVP-BHG712 plus paclitaxel. Each column represents the mean tumor weight determinations. Error bars represent SEM. *, $p < 0.05$ versus the control group.

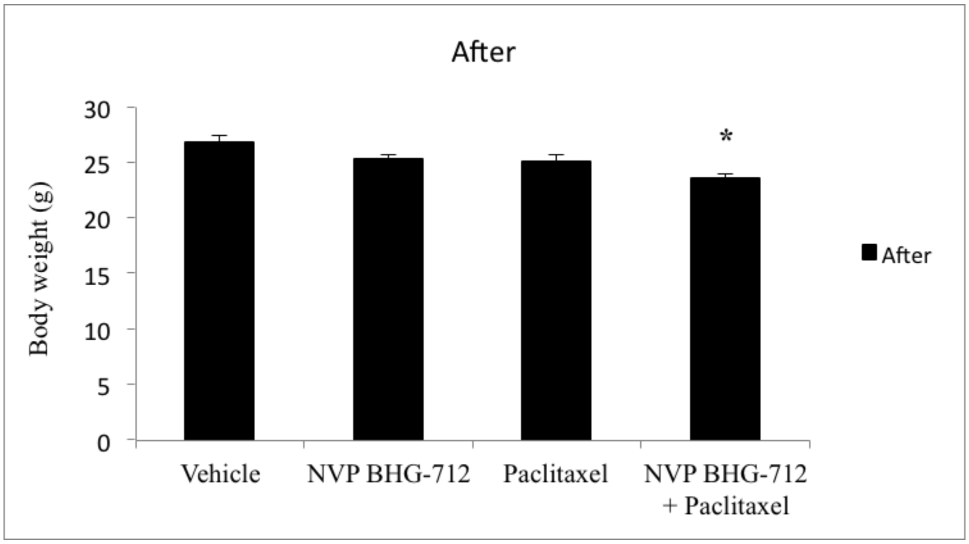

Supplemental Figure 2: The changes in mean body weight of mice (n = 8) after treatment. The mean body weight (n = 8) of the the mice treated with with vehicle, paclitaxel, NVP-BHG712 or combination of NVP-BHG712 plus paclitaxel, at the end of the 18-day treatment period. Error bars represent SEM. *: $p < 0.05$ versus vehicle group.

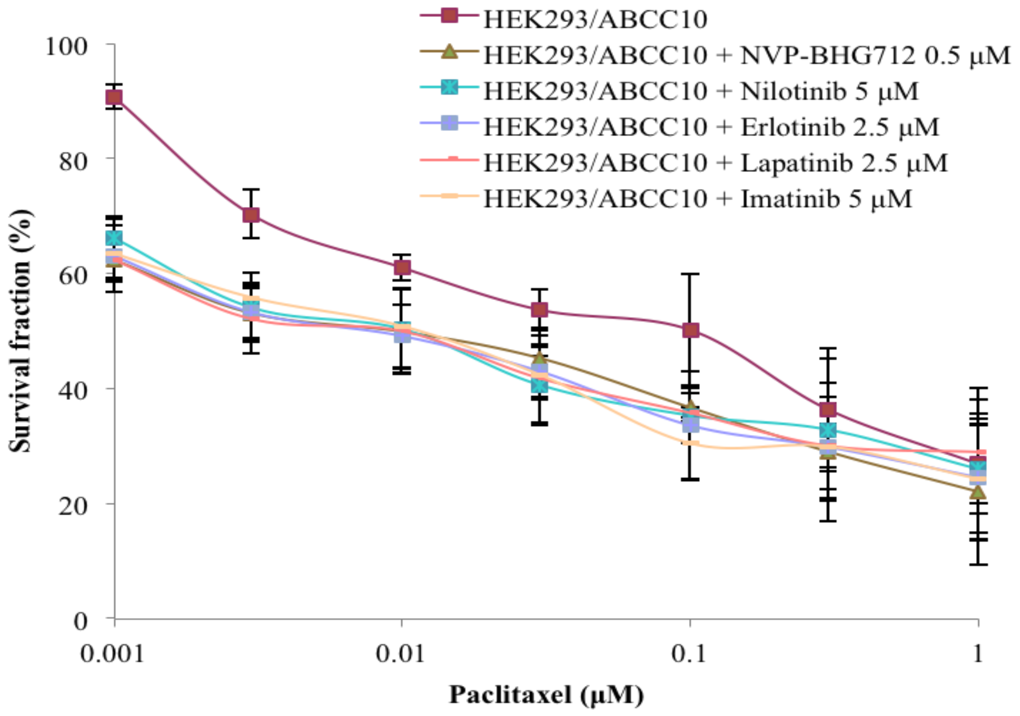

Supplemental Figure 3: Concentration-response curve for sensatization to paclitaxel with or without TKIs. The sensatization effect of TKIs in combination with paclitaxel is shown in ABCC10-expressing HEK293/ABCC10 cells. Error bars represent SD.

Supplemental Table 1: NVP-BHG712 sensitizes HEK293/ABCC10 cells to vinblastine and vincristine with no or minimal effect on HEK293/pcDNA3.1 cells

Compounds	HEK293/pcDNA3.1		HEK293/ABCC10	
	IC_{50} ± SD[a] (nM)	FR[b]	IC_{50} ± SD (nM)	FR
Docetaxel	11.01 ± 1.2	[1.0]	102.22 ± 5.0	[9.3]
+NVP-BHG712 0.25 μM	10.59 ± 0.9	[1.0]	29.60 ± 9.4**	[2.7]
+NVP-BHG712 0.5 μM	10.12 ± 1.0	[0.9]	10.63 ± 1.0**	[1.0]
+Cepharanthine 2.5 μM	10.08 ± 0.9	[0.9]	10.86 ± 1.0**	[1.0]
Vinblastine	10.21 ± 0.9	[1.0]	93.39 ± 6.4	[9.1]
+NVP-BHG712 0.25 μM	10.03 ± 0.8	[1.0]	33.78 ± 5.6**	[3.3]
+NVP-BHG712 0.5 μM	9.92 ± 0.8	[1.0]	10.66 ± 0.8**	[1.0]
+Cepharanthine 2.5 μM	10.15 ± 0.9	[1.0]	11.86± 3.3**	[1.1]

[a]IC_{50}: The drug concentration that inhibited cell survival by 50% (mean ± SD). [b]FR: fold-resistance was determined by dividing the IC_{50} values of substrate in HEK293/ABCC10 cells by the IC_{50} of substrate in HEK293/pcDNA3.1 cells in the absence of NVP-BHG712; or the IC_{50} of substrate in HEK293/pcDNA3.1 cells in the presence of NVP-BHG712 divided by the IC_{50} of substrate in HEK293/pcDNA3.1 cells in the absence of NVP-BHG712. Values in the table are representative of at least 3 independent experiments performed in triplicate. ** indicate significant statistical difference from the IC_{50} values of HEK293/ABCC10 without the reversal drug. **: p <0.01.

Supplemental Table 2: The effect of NVP-BHG712 on the cytotoxicity of paclitaxel, vinblastine and colchicine to HEK293/pcDNA3.1 and HEK293/ABCB1 cells

**Compounds**	**HEK293/pcDNA3.1**		**HEK293/ABCB1**	
	IC_{50} ± SD[a] (nM)	FR[b]	IC_{50} ± SD (nM)	FR
Paclitaxel	10.60 ± 1.3	[1.0]	2507.49 ± 424.7	[236.5]
+NVP-BHG712 0.25 μM	10.20 ± 0.9	[1.0]	2013.14 ± 443.5	[189.9]
+NVP-BHG712 0.5 μM	9.60 ± 0.5	[0.9]	1089.10 ± 399.9*	[102.7]
+Verapamil 2.5 μM	9.72 ± 0.7	[0.9]	52.05 ± 19.2**	[5.0]
Vinblastine	10.21 ± 0.9	[1.0]	937.53 ± 62.6	[91.8]
+NVP-BHG712 0.25 μM	10.03 ± 0.8	[1.0]	781.68 ± 84.4	[76.5]
+NVP-BHG712 0.5 μM	9.92 ± 0.8	[1.0]	250.27 ± 67.3**	[24.5]
+Verapamil 2.5 μM	10.15 ± 0.9	[1.0]	39.87 ± 8.4**	[3.9]
Colchicine	10.72 ± 1.6	[1.0]	787.24 ± 148.3	[73.4]
+NVP-BHG712 0.25 μM	10.24 ± 1.3	[1.0]	551.50 ± 187.6	[51.4]
+NVP-BHG712 0.5 μM	9.55 ± 1.5	[0.9]	245.95 ± 58.2**	[22.9]
+Verapamil 2.5 μM	9.61 ± 1.2	[0.9]	35.69 ± 10.3**	[3.3]

[a]IC_{50}: The drug concentration that inhibited cell survival by 50% (means ± SD). [b]FR: fold-resistance was determined by dividing the IC_{50} values of substrate in HEK293/ABCB1 cells by the IC_{50} value of substrate in HEK293/pcDNA3.1 cells in the absence of NVP-BHG712; or the IC_{50} of substrate in HEK293/pcDNA3.1 cells in the presence of NVP-BHG712 divided by the IC_{50} of substrate in HEK293pcDNA3.1 cells in the absence of NVP-BHG712. Values in table are

representative of at least three independent experiments performed in triplicate. ***** and ** indicate significant statistical difference from the IC_{50} values of HEK293/ABCB1 without the reversal drug. *: $p < 0.05$; **: $p < 0.01$.

Supplemental Table 3:. The effect of NVP-BHG712 on the cytotoxicity of vincristine to HEK293/pcDNA3.1 and HEK293/ABCC1 cells

**Compounds**	**HEK293/pcDNA3.1**		**HEK293/ABCC1**	
	IC_{50} ± SD[a] (nM)	FR[b]	IC_{50} ± SD (nM)	FR
Vincristine	9.92 ± 0.6	[1.0]	139.30 ± 17.0	[14.0]
+NVP-BHG712 0.5 μM	8.68 ± 1.4	[0.9]	43.03 ± 6.0*	[4.3]
+PAK-104P 5 μM	8.58 ± 0.2	[0.9]	8.61 ± 1.4**	[0.9]

[a]IC_{50}: The drug concentration that inhibited cell survival by 50% (means ± SD). [b]FR: fold-resistance was determined by dividing the IC_{50} values of substrate in HEK293/ABCC1 cells by the IC_{50} of substrate in HEK293/pcDNA3.1 cells in the absence of NVP-BHG712; or the IC_{50} of substrate in HEK293/pcDNA3.1 cells in the presence of NVP-BHG712 divided by the IC_{50} of substrate in HEK293/pcDNA3.1 cells in the absence of NVP-BHG712. Values in table are representative of at least three independent experiments performed in triplicate. ***** and ** indicate significant statistical difference from the IC_{50} values of HEK293/ABCC1 without the reversal drug. *: $p < 0.05$; **: $p < 0.01$.

Supplemental Table 4: The effect of NVP-BHG712 on the cytotoxicity of vincristine to HEK293/pcDNA3.1, ABCG2-482-R2, ABCG2-482-T7 and ABCG2-482-G2 cells

**Compounds**	**HEK293/pcDNA3.1**		**ABCG2-482-R2**		**ABCG2-482-T7**		**ABCG2-482-G2**	
	IC_{50} ± SD[a] (nM)	FR[b]	IC_{50} ± SD (nM)	FR	IC_{50} ± SD (nM)	FR	IC_{50} ± SD (nM)	F[illegible]
Mitoxantrone	23.29 ± 4.5	[1.0]	132.11 ± 14.1	[5.7]	482.18 ± 73.2	[20.8]	645.80 ± 47.2	[27[illegible]
+NVP-BHG712 0.25 μM	21.45 ± 5.0	[0.9]	94.58 ± 20.1	[4.0]	440.58 ± 97.9	[19.0]	386.91 ± 57.6*	[16[illegible]
+NVP-BHG712 0.5 μM	20.48 ± 1.6	[0.9]	83.99 ± 9.0*	[3.6]	403.48 ± 91.5	[17.4]	326.06 ± 52.6*	[14[illegible]
+Nilotinib 2.5 μM	26.04 ± 6.7	[1.1]	33.64 ± 14.1**	[1.5]	13.35 ± 6.7**	[0.6]	18.36 ± 5.9**	[0.[illegible]

[a]IC_{50}: The drug concentration that inhibited cell survival by 50% (means ± SD). [b]FR: fold-resistance was determined by dividing the IC_{50} values of substrate in ABCG2-482-R2, ABCG2-482-T7 or ABCG2-482-G2 cells by the IC_{50} of substrate in HEK293/pcDNA3.1 cells in the absence of NVP-BHG712; or the IC_{50} of substrate in HEK293/pcDNA3.1 cells in the presence of NVP-BHG712 divided by the IC_{50} of substrate in HEK293/pcDNA3.1 cells in the absence of NVP-BHG712. Values in table are representative of at least three independent experiments performed in triplicate. ***** and ** indicate significant statistical difference from the IC_{50} values of ABCG2-482-R2, ABCG2-482-T7 or ABCG2-482-G2 without the reversal drug. *: $p < 0.05$; **: $p < 0.01$.
